# Precise quantification of bacterial strains after fecal microbiota transplantation delineates long-term engraftment and explains outcomes

**DOI:** 10.1038/s41564-021-00966-0

**Published:** 2021-09-27

**Authors:** Varun Aggarwala, Ilaria Mogno, Zhihua Li, Chao Yang, Graham J. Britton, Alice Chen-Liaw, Josephine Mitcham, Gerold Bongers, Dirk Gevers, Jose C. Clemente, Jean-Frederic Colombel, Ari Grinspan, Jeremiah Faith

**Affiliations:** 1 Precision Immunology Institute, Icahn School of Medicine at Mount Sinai, New York, NY USA; 2 Icahn Institute for Data Science and Genomic Technology, Icahn School of Medicine at Mount Sinai, New York, NY USA; 3 Division of Gastroenterology, Icahn School of Medicine at Mount Sinai, New York, NY USA; 4 Janssen Human Microbiome Institute, Janssen Research and Development, LLC, Spring House, PA USA

**Keywords:** Microbiome, Metagenomics

## Abstract

Fecal microbiota transplantation (FMT) has been successfully applied to treat recurrent *Clostridium difficile* infection in humans, but a precise method to measure which bacterial strains stably engraft in recipients and evaluate their association with clinical outcomes is lacking. We assembled a collection of >1,000 different bacterial strains that were cultured from the fecal samples of 22 FMT donors and recipients. Using our strain collection combined with metagenomic sequencing data from the same samples, we developed a statistical approach named Strainer for the detection and tracking of bacterial strains from metagenomic sequencing data. We applied Strainer to evaluate a cohort of 13 FMT longitudinal clinical interventions and detected stable engraftment of 71% of donor microbiota strains in recipients up to 5 years post-FMT. We found that 80% of recipient gut bacterial strains pre-FMT were eliminated by FMT and that post-FMT the strains present persisted up to 5 years later, together with environmentally acquired strains. Quantification of donor bacterial strain engraftment in recipients independently explained (precision 100%, recall 95%) the clinical outcomes (relapse or success) after initial and repeat FMT. We report a compendium of bacterial species and strains that consistently engraft in recipients over time that could be used in defined live biotherapeutic products as an alternative to FMT. Our analytical framework and Strainer can be applied to systematically evaluate either FMT or defined live bacterial therapeutic studies by quantification of strain engraftment in recipients.

## Abstract (web summary)

Quantification of gut bacterial strains after fecal microbiome transplantation using the Strainer algorithm delineates long-term stable engraftment that explains patient outcomes.

## Main

Fecal microbiota transplantation (FMT)^1^ has been widely used to treat recurrent *Clostridium difficile* infection (CDI) since its superiority to vancomycin was demonstrated^2,3^. Several studies have shown that the recipient’s gut microbiota post-FMT resembles that of a healthy donor, suggesting gut microbiota restoration^4,5^. However, these studies have failed to capture the most basic principle of commensal Koch’s postulates^6,7^, which is the identification of discrete bacterial strains from the donor that are isolated as a pure culture from the cured recipient only post-FMT and not before. Eight years and tens of thousands of successful FMTs^8^ after the first successful clinical trial, there are basic but unanswered questions about how FMT alters the recipient human gut microbiota. Which strains in the FMT donor stool do or do not engraft is not clear. Which strains that colonized before FMT in recipients persist after the transplant and whether engrafting and persisting strains are durable members of the resulting microbiota is unresolved. The proportion of the recipient microbiota from the donor, recipient or environment has not been clarified. Whether any of these properties of strain engraftment and persistence predict relapse post-FMT has also not been reported.

We know that the functional impact of the gut microbiota is at the level of strains^9–13^. Therefore, quantification of gut microbiota at this resolution is essential for understanding the therapeutic potential of FMT and its impact on the health and disease of the host. Recently, several US Food and Drug Administration (FDA) advisories^14,15^ related to severe adverse events from FMT have increased safety concerns around undefined FMT, which uses the entire stool. Strain-level resolution and tracking of the transmission of culturable discrete strains during FMT could provide a route towards the application of a therapeutically defined cocktail^16^ of microbes as a safer and scalable alternative to FMT.

Achieving strain-level resolution in metagenomics has been challenging because the human gut microbiota consists of numerous bacterial strains in every species^17^, most of which have not been isolated or even detected using sequencing. As a result, previous microbiome analyses^18–20^ have focused on a lower level of resolution because finding delineating features of discrete bacterial strains, a necessary step for FMT strain tracking, is a challenge. While informative, metagenomics-only approaches^4,21,22^ require very deep sequencing to track strains using single-nucleotide polymorphisms (SNPs) in marker genes, do not model the microbiota as a defined set of discrete strains and mainly provide non-quantifiable inferences related to sharing of metagenome-assembled bacterial contigs or SNPs across FMT samples. The linking of bacterial genetic variation to a discrete unit, that is, a cultured bacterial isolate, is essential if we are to understand the transmission of strains during FMT and fulfil commensal Koch’s postulates^6,7^.

To enable precise strain tracking, we developed a high-throughput hybrid approach, where we first cultured and also sequenced the genomes of strains from FMT donors and recipients using published methods^23–25^ and then tracked these strains across metagenomic samples using the statistical method presented in this article, which we named Strainer.

## Results

### Curated, cultured and sequenced FMT strain collection

**Fig. 1:**
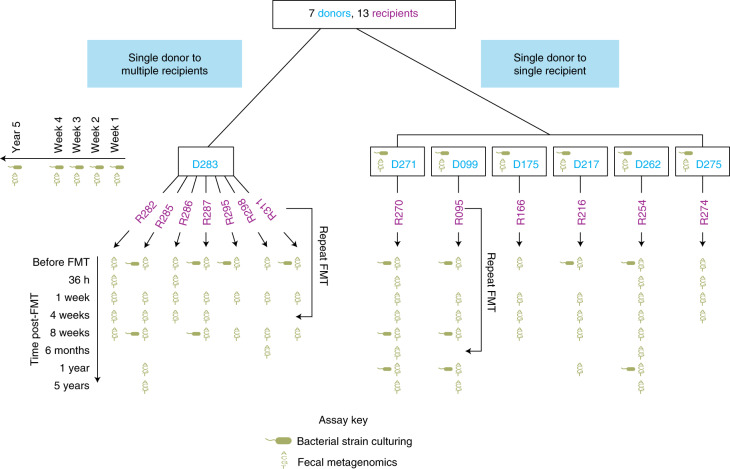
Overview of the FMT study design. Overview of the FMT study design including donors, recipients and time points when metagenomic sequencing and bacterial strain culturing was performed on fecal samples.

**Extended Data Fig. 1:**
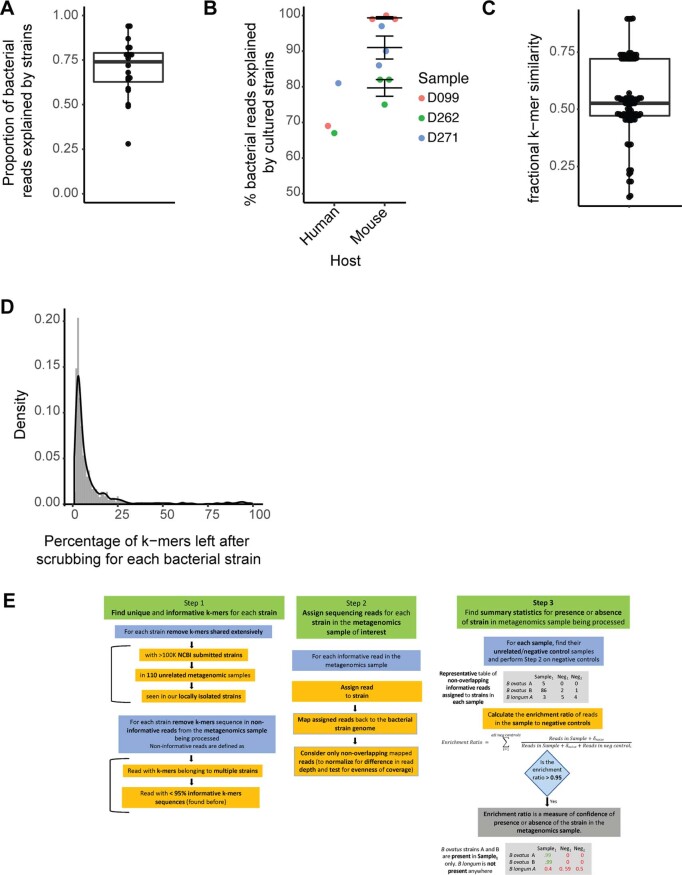
Comprehensiveness of our cultured bacterial strain library and algorithm Strainer. **a**, Proportion of bacterial reads in the metagenomics sample that are explained by the genome sequences of the cultured strain library for that sample (n = 20 biologically independent samples). Each point in the plot corresponds to a separate sample. The lower and upper bounds of the box in the boxplot corresponds to 25^th^ and 75^th^ percentile respectively, with the median line in centre. Upper whisker extends till the maxima, while the lower whisker extends till 1.5 times the inter-quartile range. Points beyond this lower limit are also plotted. **b**, Proportion of bacterial reads explained by the cultured strain library for a donor after gavaging (n = 3 independent replicates) germ-free mice with stool from (n = 3) corresponding human donors, and performing metagenomics on the mouse faecal samples. Each point corresponds to a separate sample. Data for mouse replicates for each different donor sample is presented as mean values ± SEM. **c**, Percentage similarity between (n = 96) different isolates of species *Bacteriodes ovatus* and the reference strain AAXF00000000.2. Similarity is found by comparing sequence k-mers of length 31 between genomes. Each point in the boxplot corresponds to a separate sample. The lower and upper bounds of the box in the boxplot corresponds to 25^th^ and 75^th^ percentile respectively, with the median line in centre. Upper whisker extends till the maxima, while the lower whisker extends till the minima. **d**, Proportion of bacterial reads in the metagenomics sample that are explained by the genome sequences of the cultured strain library for that sample. Each point in the boxplot corresponds to a separate sample. **e**, Overview of our algorithm Strainer. The algorithm has 3 modules, where Module-1 involves finding the unique and likely informative sequence k-mers for each strain by removing those shared extensively with unrelated sequenced strains in NCBI, unrelated metagenomics samples, and those cultured and sequenced in this study. Next, we decompose each sequencing read in the metagenomics sample of interest into its k-mers, and find reads which have k-mers belonging to multiple strains, or have <95% of informative k-mers for a single strain. We further remove these non-informative k-mers from our previous set. In Module-2 we assign sequencing reads from the metagenomics sample of interest, with a majority of informative k-mers (>95%) to each strain. Next, we map these reads to the genome of the corresponding strain, and consider the non-overlapping ones only. This step normalizes for sequencing depth across samples and checks for evenness of read distribution across the bacterial genome. Finally, in Module-3 we compare the read enrichment in a sample to unrelated samples or negative controls and present summary statistics for presence or absence of a strain in a sample. Source data

We isolated and sequenced the whole genomes of 2,987 bacterial isolates representing 1,008 unique strains (207 species) from 7 FMT healthy donors and 13 recurrent CDI FMT recipients (Table 1, Fig. 1 and Supplementary Tables 1, 8 and 9). Like our previous analyses^12,13,26^, bacterial isolates with <96% whole-genome similarity were defined as unique strains; otherwise, they were considered as multiple isolates for the representative strain. We sequenced 85 metagenomes from the 7 donor fecal samples used for FMT and recipient samples taken before and for up to 5 years after FMT. As in previous studies^27,28^, these cultured strains represented the majority of the metagenome with 70% (s.d. = 16%) of bacterial metagenomic reads mapping to the cultured strain genomes (Extended Data Fig. 1a). We also evaluated the comprehensiveness of our cultured bacterial strain library by gavaging germ-free mice (*n* = 9) with human stool and performing metagenomics on the mouse fecal samples. Our cultured bacterial strain set explained up to 90% (s.d. = 9.2%; Extended Data Fig. 1b) of bacterial reads in the gnotobiotic mice colonized with the relevant human stool, suggesting that most unexplained bacterial metagenomics reads in the human sample were from unculturable sources (for example, dead bacteria from food and environmental sources) or were systematically unable to grow in the mouse gut.

**Table 1:** Donor and recipient samples

Donor			Recipient										Success
ID	At FMT	5 years	ID	IBD	Just before FMT	36 h	1 week	4 weeks	8 weeks	6 months	1 year	5 years	
271	MC		270		MC	M	M	M	MC	M	MC	M	Y
099	MC	M	095	Y	MC		M	M	MC		M^a^, MC^a^	M	N
175	MC		166	Y	M		M	M	M				Y
217	MC		216	Y	MC		M	M	M		M^a^		N
262	MC		254	Y	MC	M	M	M	M	M	MC	M	Y
275	MC		274	Y	M	M		M	M				Y
			282		M	M	M	M	M				Y
283	MC	M	285		MC		M	M	MC		M	M	Y
			286		M	M	M	M					Y
			287		MC		M	M	MC				Y
			295		MC		M		M				N
			298	Y	M		M		M	M			Y
			311	Y	MC		M		M^a^				N

Seven donors provided their fecal material for FMT to 13 patients with either recurrent CDI or both recurrent CDI and IBD. Fecal metagenomics was performed on all stool samples. Donor strains from all donors were isolated and tracked in matching recipient metagenomes over time. Strains were also isolated from a few recipients both pre- and post-FMT. M and C indicate that metagenomics or culturing, respectively, was performed at the indicated time point. Success indicates that no relapse was noted for that patient.

^a^Sample was collected after repeat FMT (due to initial failure of FMT).

### Strainer algorithm development

The central challenge to strain tracking using metagenomics data is the identification of a set of informative sequence features, or *k*-mers, from a bacterial genome that can uniquely identify any given strain. Since each species contains many closely related distinct strains, which share a majority of genomic content^26,29^ (Extended Data Fig. 1c), identification of informative features needed to track strains is a challenge. We obtained the most informative *k*-mers (*k* = 31) to identify a strain by removing those shared extensively with bacterial genomes and fecal metagenomes from unrelated, non-cohabitating individuals, where the probability of the occurrence of the same strain is very low^26,30^ (Supplementary Table 1 and Extended Data Fig. 1d; an overview of our algorithm is shown in Extended Data Fig. 1e). We next assigned each sequencing read in a metagenomics sample to a unique strain by comparing the distribution of *k*-mers on a read with the informative *k*-mers identified earlier for that strain. Next, we mapped these assigned reads for a strain to its genome to adjust for sequencing depth and evenness of coverage. Finally, we compared the number of positionally distinct reads for a strain in the metagenomic sample with those found in unrelated samples and assigned a confidence score for the presence of that strain.

### Validation of Strainer on a defined community in gnotobiotic mice

**Fig. 2:**
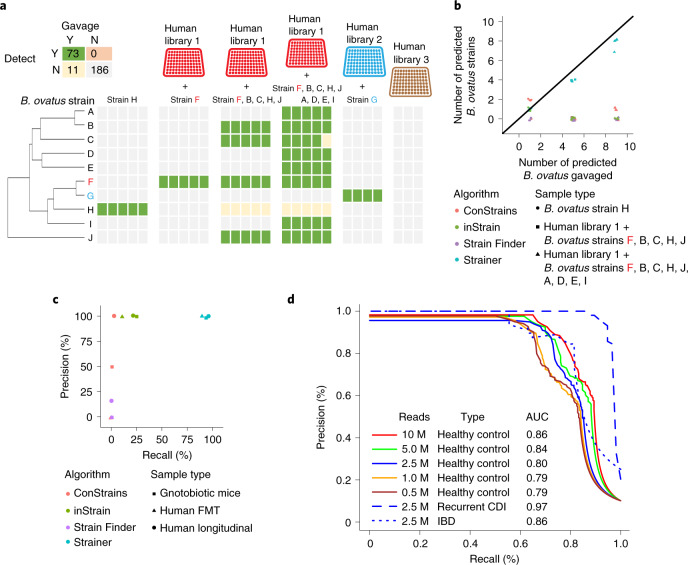
Strainer accurately detects bacterial strains in gut communities. **a**, Strainer can resolve *B. ovatus* strain(s) from other closely related strains in gnotobiotic mice. Each column represents an independent germ-free mouse gavaged with the specific *B. ovatus* strain(s) with or without a diverse human gut bacterial culture library of strains. Strains F and G were contained in human culture libraries 1 and 2, respectively. Human culture library 3 contained no *B. ovatus*, while the remaining *B. ovatus* isolates were isolated from other human fecal samples. The green box indicates that the strain was introduced in the mice and detected in metagenomics (true positive); grey indicates that the strain was not detected (true negative); orange indicates the strain was detected but was not introduced (false positive); yellow indicates that the strain was not detected but was gavaged in the mice (unknown since gavaging a strain does not always lead to stable colonization). **b**, Performance of the SNP-based inference strain detection algorithms ConStrains, Strain Finder, inStrain and our Strainer approach on detecting the number of *B. ovatus* strain(s) in gnotobiotic mice. **c**, Precision–recall curves to assess the performance of SNP-based inference strain tracking approaches and Strainer on real datasets ranging from sequential gavaging of a defined set of strains in gnotobiotic mice, FMT donor recipient pairs and tracking strain stability in a healthy individual over time. **d**, Performance assessment of Strainer’s ability to match strains to the metagenome of the sample from which they were isolated. The solid lines denote the results at different sequencing depths after application of our algorithm on 261 strains isolated from healthy controls. Blue indicates the sequencing depth of 2.5 million (M) reads, while the dashed line indicates the result after application of Strainer on 56 strains isolated from patients with recurrent CDI. The dotted curve is for 54 strains from patients with IBD. The AUC of the precision–recall curves is shown in the figure. Source data

We tested the ability of Strainer to accurately detect bacterial strains in gnotobiotic mice sequentially gavaged with defined culture collections of bacteria isolated from three different human fecal samples^13^ and a subset of ten unique strains of the common human gut commensal bacterium *Bacteroides ovatus* (Fig. 2a and Supplementary Table 2). We quantified our overall performance in these simplified communities using precision and recall, which were 100% and 86.9%, respectively, with no false positives in 280 different tests (specificity 100%).

### Benchmarking of Strainer

Our dataset of strains isolated from matched and metagenomically sequenced FMT samples provides an in vivo experimental benchmark for rigorous comparison of SNP-based inference approaches for tracking SNP strain proxies in metagenomics. We tested the previously published Strain Finder^4^, ConStrains^21^ and inStrain^22^ algorithms on our gnotobiotic mice dataset. These SNP proxy algorithms, which were developed on simulated and in vitro datasets, have an additional challenge beyond our reference-based method in that they must first infer the strains from the metagenomes themselves. Any strain not inferred would lead to false negatives across the dataset and any strain incorrectly inferred will propagate false positives in any sample where it is falsely detected. Therefore, we first tested the ability of each algorithm to estimate the correct number of *B. ovatus* strains in each mouse. However, all algorithms struggled to do so, while Strainer’s detection was largely in line with the number of *B. ovatus* strains gavaged into the mice (Fig. 2b, *R*^2^ = 0.99 for Strainer). We then tested if these inferred strains matched with those gavaged to the germ-free mice. To do so, we provided the raw unassembled sequencing reads (approximately 2.1 million) for every strain (from its pure culture) as a distinct metagenomic ‘truth’ sample and determined if any of the algorithms could match these unassembled strain reads with the correct metagenomics sample. None of these algorithms were able to do so (Fig. 2c) but inStrain could correctly identify the unassembled reads from different strain genomes as distinct. In this study, the sensitivity of the ConStrains and Strain Finder algorithms was 0, while for inStrain it was 10.1 and our approach was 88.7. None of the algorithms had false positives.

### Strainer validation on complex human gut microbiotas

To evaluate Strainer in a more realistic and challenging scenario, we next tested it in the context of several complex human gut microbiota communities with high species overlap but little-to-no strain overlap. This resembles the use-case application for FMT where a potentially transmitted bacterial strain has to be precisely detected across multiple individuals, while differentiating it from other related commensal strains from the same species. We sequenced the fecal metagenome of 10 unrelated individuals as well as the genome of 261 bacterial strains isolated from the same fecal samples (Supplementary Table 3) and then evaluated the ability of Strainer to detect these strains in the correct individual’s metagenome, while not falsely detecting it in the other 9 other samples. With 10 million metagenomic reads per sample, we reached a precision of 93.9% at a recall of 72.4% with an area under the curve (AUC) of 0.86 (Fig. 2d). Although we attained slightly higher recall with deeper metagenomics, even 500,000 metagenomic sequencing reads were sufficient to reach a precision of 95.8% with a recall of 57.6% (Fig. 2d). We generated similar testing datasets (Supplementary Tables 4 and 5) from five individuals with recurrent CDI and four with irritable bowel disease (IBD) and found slightly higher AUC for recurrent CDI as a result of the low diversity of the gut microbiome in recurrent CDI^31^ (Fig. 2d). While we report high overall AUC, some taxonomic orders were easier to detect than others (Supplementary Table 6) with performance suffering for species with small numbers of available reference genomes to infer informative *k*-mers from and for species isolated by highly selective culture enrichments where culture is more sensitive than metagenomics^23^. Altogether, these results demonstrate that our algorithm can accurately track sequenced bacterial strains in a metagenome, thus allowing quantification of discrete donor strain transmission in FMT.

### FMT strain dynamics in patients with recurrent CDI

In a clinical cohort^32^ (overview of the FMT study design in Fig. 1 and Table 1), six FMT donors each provided their sample to a single recipient, which was sampled at multiple time points post-FMT, while one donor provided the sample to seven different patients.

**Fig. 3:**
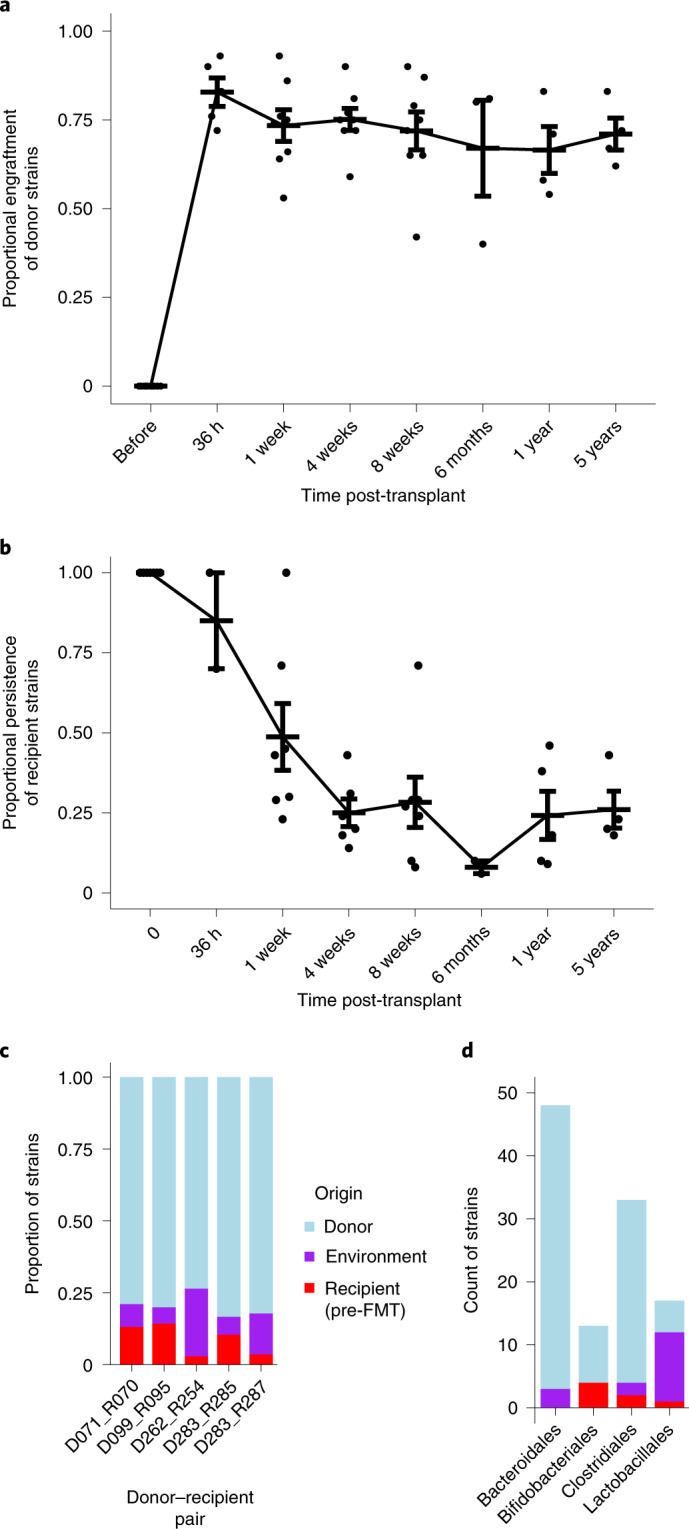
FMT strain dynamics in recipients for up to 5 years. **a**, Strains from the donor (*n* = 6 biologically independent samples) remained stably engrafted in successful post-FMT patients (*n* = 13 biologically independent samples) for at least 5 years after transplant. Data at each time point are presented as mean values ± s.e.m. **b**, Strains isolated from a recipient (*n* = 7 biologically independent samples) before FMT were rapidly lost with a small proportion persisting at longer timescales. Data at each time point are presented as mean values ± s.e.m. **c**, Proportion of donor, recipient and environmental strains detected in patients post-FMT. Environmental strains are non-donor and non-recipient (before FMT) in origin, which are both cultured and metagenomically detected post-FMT. **d**, Count of strains detected in patients post-FMT subclassified by major phylogenetic taxa (at order level) and coloured based on their origin. Source data

**Extended Data Fig. 2:**
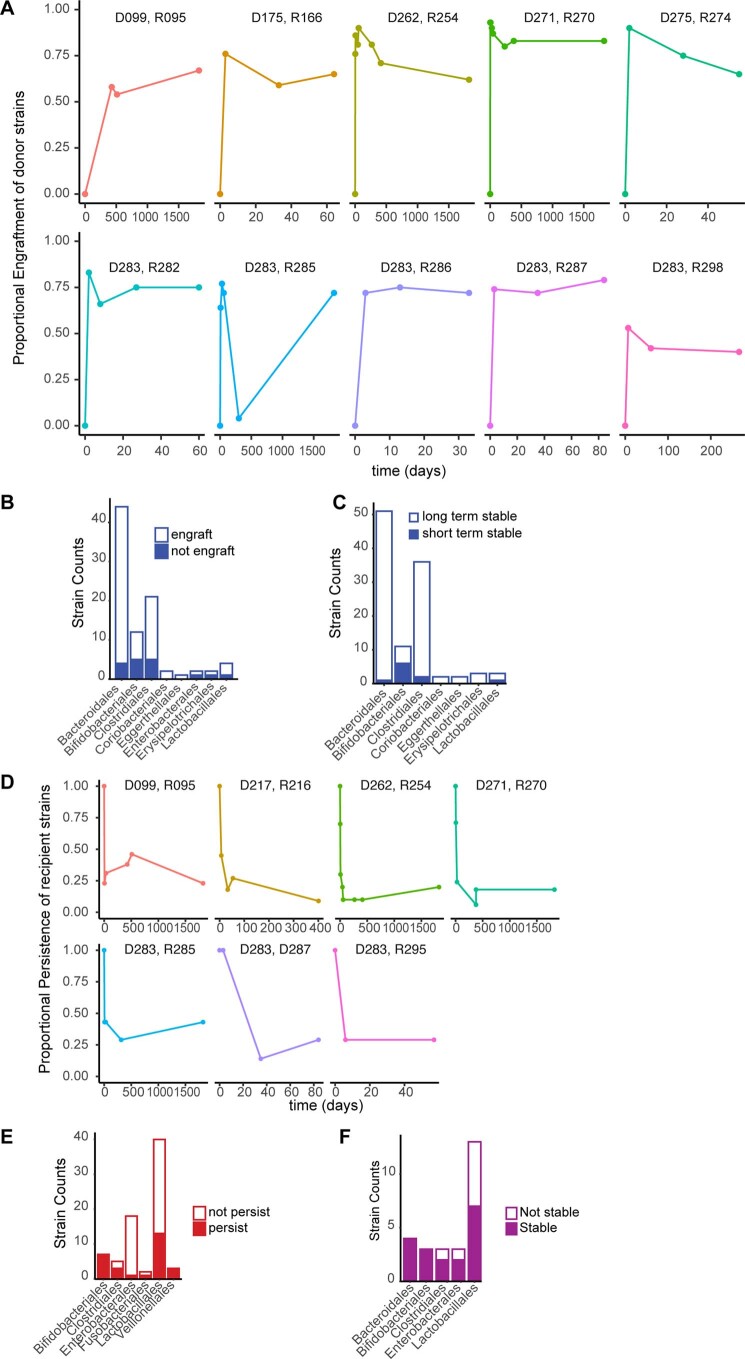
FMT strain dynamics (donor, pre-FMT recipient and environmental strains) in recipients post-FMT. **a**, Trajectory of proportional strain engraftment of donor strains in each recipient at all available timepoints (in days). The donor recipient pair ids are at the top of each plot. **b**, Number of strains that transmit and engraft for at least 8-weeks in patients post-FMT (single FMT donor to recipient setting) grouped by taxonomic order. **c**, The number of strains colonized at 8 weeks (short term) that engraft for at least 6-months or more (long-term) in patients post-FMT (both single FMT donor to single and multiple recipients setting) grouped by taxonomic order. **d**, Trajectory of proportional persistence of recipient’s strains post-FMT at all available timepoints (in days). The donor recipient pair ids are at the top of each plot. **e**, The number of the recipient’s original strains that persist for at least 8-weeks post-FMT, grouped by taxonomic order. **f**, The number of environment strains (that is non-donor and non-recipient in origin) that engraft in patients stably over multiple timepoints (>1 week) post-FMT, grouped by taxonomic order. Source data

Previous approaches have demonstrated sharing of microbiota between donor and recipient post-FMT in recurrent CDI but precise quantification of engraftment is still unresolved. We used Strainer to measure the engraftment of donor strains in the recipients and defined the proportional engraftment of donor strains (PEDS) metric as the number of donor strains detected in a recipient post-FMT divided by the total number of strains isolated from the donor. We tracked ten non-relapsed recipients for up to five years after FMT and found consistent and stable engraftment of donor strains (Fig. 3a and individual trajectories for donor–recipient pairs in Extended Data Fig. 2a). In these individuals, we report an average engraftment of 83% (s.d. = 9%) at 36 h, which stabilizes at 71% (s.d. = 16%) at 8 weeks (a commonly noted clinical end point for measuring efficacy) and is consistently high at 71% (s.d. = 9%) even 5 years later, demonstrating that gut microbiota manipulation by FMT can lead to a near permanent engraftment of a stable^26^ set of donor bacterial strains in patients with recurrent CDI. Strains belonging to the order Bifidobacteriales engrafted less at 8 weeks (67% of strains), while strains in the order Bacteriodales engrafted higher (92% of strains; Extended Data Fig. 2b); we observed very little engraftment from the order Lactobacillales.

We found 50 out of 51 strains belonging to the order Bacteriodales, which engrafted at 8 weeks to remain stably engrafted at a longer timescale of 6 months or more (Extended Data Fig. 2c). However, fewer strains belonging to the order Bifidobacteriales, which engrafted at 8 weeks, remained stably engrafted at 6 months or longer timescale (only 5 out of 11, *P* < 10^−5^, Fisher’s exact test).

### Validation of bacterial strain engraftment by culturing

The isolation and sequencing of strains from both donor and recipient post-FMT represents validation of the commensal Koch’s postulates^6,7^. Demonstrating transmission of donor bacterial strains from multiple species and across different FMT interventions by culture^33^ would also provide additional proof of engraftment besides validation of commensal Koch’s postulates. Therefore, we also cultured strains from five recipients both pre- and post-FMT (Fig. 1a) and compared the strain composition to that of the donor to experimentally validate bacterial strain transmission. We never isolated a donor strain in any recipient before FMT; however, post-FMT we isolated 48 donor strains in recipients consisting of 16 different species (Supplementary Table 7). If we quantify algorithmic strain tracking performance on these criterion standard strains isolated in both donor and recipient using different algorithms, our approach for FMT tracking had an overall sensitivity of 92.9 (with 1 false positive) while inStrain had 25.3, Strain Finder had 0 and ConStrains had 1.4 (Fig. 2c). For tracking in longitudinally cultured samples, that is, in a healthy individual across multiple time points, our algorithm had an overall sensitivity of 96.6 (with no false positive) while inStrain had 21.8, Strain Finder had 0 and ConStrains had 3.4. This comparison on human, experimentally verified strain transmission datasets demonstrates that Strainer is superior to existing approaches.

### Original resident strains are replaced by FMT

Several studies have shown that resident microbiota strains create ecological niches^34,35^, which in turn can influence the engraftment of donor bacteria post-FMT. Thus, it is crucial to identify the bacterial strains present pre-FMT and resolve their persistence dynamics after transplantation. We isolated and sequenced the pre-FMT resident strains in seven recipients and tracked them for up to five years in each recipient’s metagenome. Similar to the PEDS metric, we defined proportional persistence of recipient strains (PPRS) as the ratio between the strains of the recipient observed post-FMT and the total recipient strains cultured pre-FMT. Unlike the rapid high engraftment of donor strains, we found a more graduated decline in the PPRS (Fig. 3b and individual trajectories for donor–recipient pairs in Extended Data Fig. 2d) with the overall persistence decreasing to 49% (s.d. = 28%) at 1 week and 21% (s.d. = 10%) at 8 weeks (*P* < 0.02, Wilcoxon test). The recipient strains belonging to the order Bifidobacteriales always persisted (seven of seven) in the recipients for eight weeks post-FMT (Extended Data Fig. 2e). However, recipient strains from the orders Lactobacillales and Enterobacterales were largely eliminated by the FMT. As in previous studies^36^, we mostly observed an instability of the recipient gut microbiota; however, our approach demonstrates that a subset of the original strains remain durably colonized.

### Engraftment of non-donor strains after FMT

We next investigated niche occupancy of donor and pre-FMT recipient strains in the gut. We isolated and tracked strains from 5 individuals post-FMT; we found 24 strains that were non-donor and non-recipient in origin, which were metagenomically detected and cultured in recipients post-FMT. On average, in a post-FMT patient, 8.9% of strains persisted from the recipient pre-FMT, 79.6% strains engrafted from the donor and 11.5% strains were non-donor or non-recipient in origin (Fig. 3c). Although their origin and mode of transfer is unknown, these environmental strains belong to phylogenetic taxa detected in both healthy donors and recipients before FMT (Fig. 3d) with similar colonization patterns (Extended Data Fig. 2f). These results suggest that approximately 11.5% of the recipient niche space was stably colonized by other sources and that defined live biotherapeutic products (LBPs) with more limited niche occupancy will require a larger acquisition of environmental microbes for the host to become fully colonized. Alternatively, these organisms may represent strains that were previously below the limit of detection or culturing in the donor and recipient before the transplant, although this seems unlikely to be the case for all strains since many are from taxonomic groups (Fig. 3d) where our precision and recall were very high in our benchmarks (Supplementary Table 6).

### Donor engraftment explains recurrent CDI FMT clinical outcomes

**Fig. 4:**
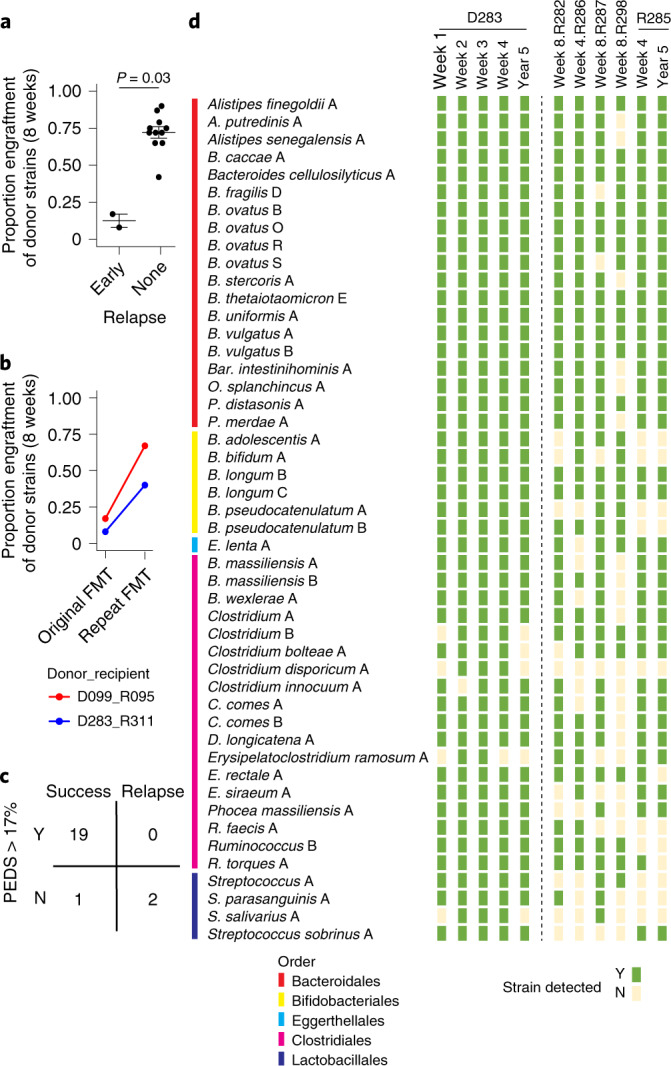
Donor engraftment explains recurrent CDI FMT clinical outcomes. **a,** PEDS at 8 weeks can predict early relapse of FMT in patients (*n* = 13 biologically independent samples) with recurrent CDI. A two-sided Wilcoxon test was used to estimate statistical significance. **b**, The PEDS metric can elucidate the successful outcome of repeat FMT in patients who relapsed with recurrent CDI after the initial FMT. **c**, Predictive power of our approach on all available FMT samples where clinical evaluation was independently noted. Whenever we reported clinical success we found engraftment to be above the threshold of 17% (*n* = 19 true positives) with 1 false negative. Clinical relapse was always independently associated with low engraftment (*n* = 2 true negatives) with no false negatives. **d**, Bacterial strain engraftment and identification of highly transmissible strains that stably engraft in multiple recipients. The first four columns are weekly metagenomic samples from the donor, while the fifth column is the donor sample from five years later. The next six columns are from the FMT recipients who did not have an early relapse. The last column is from one of the recipients five years later. Strainer was used to find the presence (green) or absence (yellow) of each bacterial strain from the corresponding metagenomics sample. Source data

FMT interventions typically evaluate the efficacy of the treatment at the eight-week time point post-FMT by comparing the number of patients that achieved the clinical end point with those who failed to do so. PEDS provides a potential quantitative surrogate marker to understand FMT clinical success or relapse. In the two patients in this cohort who had an early relapse within 8 weeks of FMT, we found significantly reduced PEDS (Fig. 4a, *P* = 0.03, two-sided Wilcoxon test) compared to those that successfully achieve the clinical end point of no CDI recurrence at 8 weeks post-FMT. This suggests that precise engraftment of donor strains in recipients can independently explain the early clinical outcome of an FMT intervention since individuals could be perfectly classified into relapse or non-relapse with a PEDS threshold of 17%.

Empirical clinical results suggest that individuals who undergo repeat FMT often respond the second time. Next, we evaluated if our PEDS metric could elucidate the outcome of repeat FMT in these patients. The two recipients (R095 and R311) who had an early failure, received a repeat dose of FMT and reported clinical success (that is, no relapse with recurrent CDI recurrence) at future time points (including at five years for R095). PEDS was notably higher after the repeat dose (Fig. 4b).

Since PEDS was able to explain both relapse and outcome of repeat FMT in patients, we evaluated the overall predictive power of our approach on all available FMT samples where clinical evaluation was independently noted (Fig. 4c). Whenever we reported clinical success (that is, no relapse), we found engraftment to be above the threshold of 17% (*n* = 19 true positives) with 1 false negative. Similarly, clinical relapse was always independently associated with low engraftment (*n* = 2 true negatives) with no false negatives. Together, these results suggest that engraftment of donor strains at any time point is an accurate and robust metric (precision = 100%, sensitivity = 95%) to independently explain the clinical outcome of FMT, both for initial FMT and after a repeat FMT. However, the reasons for lower engraftment resulting in unsuccessful FMT in patients are still unclear.

**Extended Data Fig. 3:**
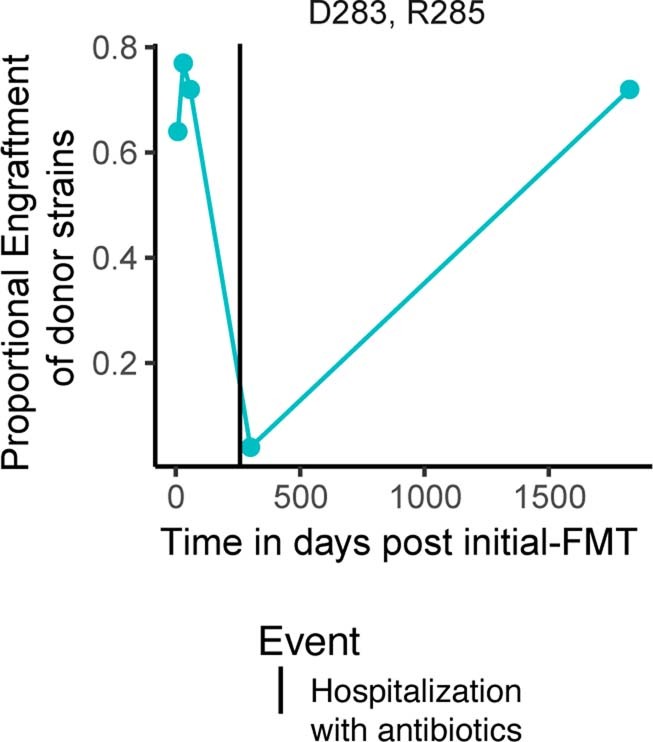
Clinical implications. Engraftment of donor D283 strains in recipient R285, which did not relapse but rather had a temporary loss in detectability of the donor strains during antibiotic treatment for severe diarrhoea. Source data

The one case of very low engraftment in an otherwise successful FMT with no relapse occurred in patient R285 (Extended Data Fig. 3). This patient reported high engraftment of 77% at day 30, 72% at day 58, reduction to 4% on day 300 and an increase again 5 years later to 72%. The patient was symptom-free at both two months and five years, in sync with expectations due to higher engraftment at those time points, which is why the low engraftment was initially surprising. However, this patient was hospitalized with severe diarrhoea and antibiotics on day 258 post-FMT, which perhaps explains the low PEDS measured in their metagenome on day 300, although this would suggest that their microbiome had not recovered over a relatively substantial period of 42 d. Importantly, this individual was not given a repeat FMT, suggesting that the lower engraftment at day 300 post-FMT resulted in the large majority of engrafted strains being reduced below the detection limit of our algorithm but impressively not being eliminated from the gut.

### Selection of bacterial strains for defined live bacterial therapeutics

Precision analysis of FMT to identify culturable distinct strains that can be used to treat recurrent CDI provides a route to selection of a minimal cocktail of bacteria for use in defined LBPs as a safer and scalable alternative to FMT. Using our analytical approach and Strainer, we identified the culturable engrafting fraction of human-tested donor fecal microbiotas and strains that did not engraft. Defined LBPs are important because they are more scalable than FMT and reduce the chances of transmission of multi-drug-resistant organisms to recipients, a possibility that cannot be excluded with regular FMT. Donor D283 was used for multiple (*n* = 5 non-relapsing) recipients and therefore provides us with more power to detect engraftment consistency of single strains (Fig. 4d). Focusing on the highly transmissible strains that stably engraft in at least 4 out of 5 non-relapsing recipients from D283, we found that those belonging to the order Bacteriodales always engrafted (100%, 19 out of 19, even up to 5 years), suggesting that these strains and others that stably engraft for longer in successfully treated patients, might be utilized for LBPs. We provide a compendium of species from all donors and the frequency at which strains from each species engraft in recipients (Table 2). These engrafting strains and species provide validated components to select for use in future trials.

**Table 2:** Compendium of frequently engrafting species

Species	Number of donors	Number of strains cultured	Number of recipients transferred to	Number of strains engrafted in recipients	Engraftment efficacy
*Bacteroides ovatus*	6	10	34	30	0.88
*Bacteroides vulgatus*	6	13	25	25	1
*Bifidobacterium longum*	6	9	21	14	0.67
*Bacteroides uniformis*	7	7	13	13	1
*Bacteroides thetaiotaomicron*	6	7	13	12	0.92
*Ruminococcus obeum*	3	4	16	12	0.75
*Parabacteroides distasonis*	5	5	12	11	0.92
*Coprococcus comes*	3	4	16	10	0.63
*Bacteroides fragilis*	5	5	11	9	0.82
*Dorea longicatena*	5	5	11	9	0.82
*Parabacteroides merdae*	4	4	10	9	0.9
*Bacteroides cellulosilyticus*	3	4	10	9	0.9
*Bifidobacterium pseudocatenulatum*	4	5	17	8	0.47
*Odoribacter splanchnicus*	3	3	9	8	0.89
*Ruminococcus torques*	3	3	9	8	0.89
*Bacteroides caccae*	2	2	8	8	1
*Alistipes putredinis*	2	2	8	7	0.88
*Alistipes onderdonkii*	2	2	8	7	0.88
*Eubacterium rectale*	2	2	8	7	0.88
*Collinsella aerofaciens*	3	6	6	6	1
*Blautia massiliensis*	2	2	8	5	0.63
*Bacteroides stercoris*	1	1	7	6	0.86
*Barnesiella intestinihominis*	1	1	7	6	0.86
*fsenegalensis*	1	1	7	6	0.86
*Bifidobacterium adolescentis*	5	5	11	5	0.45
*Eggerthella lenta*	2	2	8	5	0.63
*Clostridium ramosum*	2	2	8	4	0.5
*Bifidobacterium bifidum*	2	2	8	4	0.5
*Blautia wexlerae*	2	3	8	6	0.75
*Clostridium leptum*	1	1	7	4	0.57
*Streptococcus parasanguinis*	1	2	14	4	0.29
*Eubacterium siraeum*	2	2	8	3	0.38
*Streptococcus salivarius*	2	2	8	3	0.38
*Roseburia faecis*	1	1	7	3	0.43
*Bacteroides intestinalis*	4	4	4	2	0.5
*Escherichia coli*	4	5	5	2	0.4
*Bacteroides clarus*	2	2	2	2	1
*Bacteroides xylanisolvens*	2	2	2	2	1
*Parabacteroides johnsonii*	2	2	2	2	1
*Anaerotruncus colihominis*	2	2	2	2	1
*Bacteroides massiliensis*	2	2	2	2	1
*Alistipes shahii*	2	2	2	2	1

Identification of a set of bacterial species for LBP, based on their culturing and engraftment efficacy across recipients. ‘Number of donors’ corresponds to the donors where strains from this species were cultured or detected metagenomically. ‘Number of strains cultured’ represents the unique strains cultured and detected metagenomically for this species. ‘Number of recipients transferred to’ corresponds to the number of FMT recipients (counted separately for each strain cultured from this species), which received a strain from this species. ‘Number of strains engrafted in recipients’ represents the strains that engrafted for at least eight weeks (a common clinical end point) in a recipient. ‘Engraftment efficacy’ was calculated as the ratio of ‘Strain engraftment’ (column 5) and ‘Recipients transferred to’ (column 4). In this study, we only consider bacterial species for which two or more strains were engrafted in recipients.

## Discussion

We developed the Strainer algorithm to track sequenced bacterial strains in metagenomic sequencing datasets. In combination with high-throughput strain culturing and metagenomic sequencing of 13 donor and recipient pairs over multiple time points, we rigorously benchmarked existing algorithms and showed that most FMT donor strains (>70%) and a minority of recipient strains (<25%) are retained for at least 5 years after FMT in non-relapsing patients. These results suggest that FMT represents a semipermanent alteration of the host microbiome—a remarkably durable therapeutic from a single administration, whose stability resembles that of healthy controls. We showed that PEDS is a predictive measure of FMT success and recurrent CDI relapse. While successful FMT is associated with high engraftment of donor strains, this engraftment is enabled by strains from a subset of taxonomic groups that engraft very well, in line with previous studies^37^, and suggests that a specific group of strains determines the long-term outcome of FMT interventions.

We acknowledge that our approach is dependent on the sequencing of cultivated bacterial strains, which it detects and tracks from the metagenomes. Approaches for high-throughput strain culturing and isolation are improving^23–25^ but are still limited by factors of cost, time and difficulty in culturing microorganisms from some species. Our approach is best suited to therapies that use one donor or a defined LBP for many recipients. This will likely be the dominant use-case if defined LBPs with equivalent efficacy to FMT are developed for different therapy areas. In addition, the in vivo strain genome and metagenome benchmarks in our study, combined with the rapidly increasing number of bacterial genomes to better define the pangenome of each species, could result in the development of improved algorithms or parameters for culture-independent strain tracking^16^.

The quantitative nature of our framework can be used to inform gut microbiota manipulation study designs beyond clinical efficacy. For example, if oral FMT capsules result in similar engraftment and outcomes as colonoscopic infusion of donor material^38,39^, future study designs might rely on a less invasive oral administration. Some FMT trials^40,41^ use multiple transplants over many weeks and our framework and algorithm could accurately evaluate the engraftment gains to determine the cost/benefit of repeated doses.

Recently, the FDA^14,15,42^ issued multiple safety alerts regarding the use of FMT and risk for serious adverse events due to transmission^43^ of multi-drug-resistant organisms in the fecal material used for FMT. An important result from our study is the identification of a select mixture of live bacterial strains that stably engraft for five years in patients who are symptom-free post-FMT. These strains are an ideal starting point for a synthetic LBP-free of multi-drug-resistant organisms that might serve as an FMT alternative for recurrent CDI.

## Methods

### Germ-free mice and colonization with cultured bacteria

The mice experiments were performed as a part of other published studies to understand the strain-level differences and their role in fecal IgA levels^12,13,31,44^, as well as the impact of the IBD and non-IBD microbiome on baseline immune tone and colitis. These data were analysed in this study to evaluate the performance of strain tracking algorithms in defined communities of bacterial strains in gnotobiotic mice. They were also used to determine the proportion of a human fecal community represented by our isolated and sequenced bacterial genomes in a separate environment that would only propagate (a subset of) the viable microbial members in a human metagenome.

### Human participants

All individuals in the study were aged 18 and over. Written consent was obtained from all individuals recruited in the study using a protocol approved by the Mount Sinai Institutional Review Board (HS no. 11-01669). Donors and patients who received FMT for recurrent CDI or recurrent CDI and IBD were described in a previous study analysed with 16S ribosomal RNA amplicon sequencing^32^. This study is a longitudinal observational study and we only considered the subset of individuals for which donor and recipient stool samples (from multiple time points) had been collected.

### Fecal sample collection, DNA extraction and shotgun metagenomic sequencing

We followed the protocol previously described in our published^12,13,31^ studies. Briefly, samples were aliquoted on dry ice or liquid nitrogen and stored at −80 **°**C. DNA was then extracted by bead beating in phenol chloroform. Illumina sequencing libraries were generated from sonicated DNA, ligation products purified and finally enrichment PCR was performed. Samples were pooled in equal proportions and size-selected before sequencing with an Illumina HiSeq (paired-end 150 base pairs (bp)). The sequence data files (FASTQ) for all metagenomic sequencing samples are stored in the Sequence Read Archive (SRA) under project number PRJNA637878.

### High-throughput anaerobic bacterial culture

We utilized a well-established, robotized platform that enables isolation and culturing of a high proportion of bacteria found in the human gut^12,13,26,44^. Briefly, the steps involved first plating clarified stool samples on solid media. This was followed by growth under a range of environmental conditions designed to cultivate anaerobic, microaerophilic, aerobic and spore-forming bacteria. Next, 384 colonies were picked for each donor sample and regrown in liquid media in multi-well plates. Each isolate was then identified by a combination of matrix-assisted laser desorption/ionization-time of flight mass spectrometry, 16S ribosomal DNA and whole-genome sequencing. Using this knowledge, the original 384 isolates were de-replicated and unique strains for each donor were archived in multi-well plates, which allowed for automated selection of specific strains and subcommunities. The sequencing reads from each culture isolated were quality-filtered with Trimmomatic^45^ and assembled using SPAdes^46^.

### Strains as bacterial isolates with <96% similarity

Like our previous analyses^13,26,44^, bacterial isolates with <96% whole-genome similarity were defined as unique strains, otherwise they were considered as multiple isolates for the representative strain. Pairwise strain similarity was found with the *k*-mer counting software KMC v.3.0 (ref. ^47^). Cultured strains are available on request.

### Strainer framework for the detection of bacterial strains from metagenomic samples

The algorithmic framework Strainer has 3 separate parts (Extended Data Fig. 1e); the first part involves finding the unique and likely informative sequence *k*-mers (*k* = 31) for each strain by removing those shared extensively with unrelated >100,000 bacterial sequenced strains in the National Center for Biotechnology Information, unrelated 116 metagenomics samples^48,49^ from Russia and Mongolia and >1,000 bacterial genomes cultured and sequenced in this study. Any *k*-mer shared extensively (beyond a threshold) between these unrelated samples was removed from the original set of informative *k*-mers. However, some bacterial species shared genomic sequences extensively and this stringent criterion was relaxed (that is, the sharing cut-off was iteratively increased) until we had at least 4% of genomic *k*-mers left for each bacterial strain. Next, we decomposed each sequencing read in the metagenomics sample of interest into its *k*-mers and found reads that had *k*-mers belonging to multiple strains or had <95% of informative *k*-mers for a single strain. We further removed these non-informative *k*-mers from our previous set. In the second part, we assigned sequencing reads from the metagenomics sample of interest, with most informative *k*-mers (>95% found at the end of part 1) to each strain. Next, we mapped these reads to the genome of the corresponding strain (Bowtie version 2.4.1 (ref. ^50^) with the very sensitive, no-mixed, no-discordant options) and considered the non-overlapping ones only. This step normalizes for sequencing depth across samples and checks for evenness of read distribution across the bacterial genome. Finally, in the last part, we compared the read enrichment in a sample to unrelated samples or negative controls and presented summary statistics for the presence or absence of a strain in a sample. We did not find substantial differences in algorithm performance for *k*-mer sizes around this range.

Further details, the software and a demo application are available at https://bitbucket.org/faithj02/strainer-metagenomics/.

### Comparison with other SNP-based inference approaches for strain tracking

We performed comparison of our algorithm with three popular strain tracking algorithms—Strain Finder^4^, ConStrains^21^ and inStrain^22^—on experimental datasets ranging from a defined set of strains in gnotobiotic mice, FMT donor–recipient pairs and tracking the strain stability in a healthy individual over time. These algorithms were run in the default setting with standard databases (AMPHORA2 provided with StrainFinder and UHGG v.1 by inStrain). Samtools v.1.11 and Bowtie v.2.2.8 were uniformly used for these approaches.

The first goal of each of these approaches was to infer the unique set of strains for each species in a metagenome. In this setting, we only provided the metagenomics samples (in gnotobiotic mice where the maximum number of strains from a species was known) and queried if these approaches could predict the right number of strains in a sample. In this study, StrainFinder failed to predict anything because their dependent database (AMPHORA) did not include the common commensal species *B. ovatus*.

Next, we tested if these predicted strains matched with those gavaged to the germ-free mice or isolated and cultured from a human sample. We provided the raw unassembled sequencing reads (approximately 2.1 million) for every strain genome (from its pure culture) as a distinct sample. These algorithms aim to detect bacterial strains but they do not provide the inferred genomic sequence of such strains to compare to the criterion standard truth; instead, we checked if any of these algorithms could cluster or matched the unassembled criterion standard true strain reads with the correct metagenomics sample. These data should also simplify the challenge because the raw unassembled reads for each strain have excess sequencing coverage of all SNPs, thus making the SNP-based inference process easier. In this study, we assessed the accuracy of prediction by considering a match to be the true positive if the unassembled strain reads clustered with the correct metagenomics sample(s), false positive if it clustered to an incorrect sample (negative control or unrelated individual) and false negative as the inability to cluster the strain reads with the correct metagenomics sample.

All the raw data (matched metagenomics samples, genomic sequence of the bacterial strains and their unassembled sequenced reads) used for comparison and the exact commands run from different strain tracking packages are also available on our software biobucket repository.

### Statistical analysis and plotting

Precision–recall curves were plotted with the R package PRROC v.1.3.1. Analysis was performed in Python v.2.7.16 and RStudio v.1.1.453. The radial graphs for phylogeny were plotted using the R packages ape v.5.4 and phytools v.0.7. Our statistical framework, associated databases and code are publicly available. We provide two different test scenarios with relevant metagenomics and bacterial strain samples to help the user understand and execute our algorithmic approach on their customized data. The authors would enthusiastically respond to all reasonable requests for customization of the Strainer code and statistical framework.

### Metagenomics and comprehensiveness of isolated bacterial strains

We sequenced an average of approximately 5.2 million reads from a total of 85 fecal gut metagenomics samples. The cultured strain library from the fecal gut samples was comprehensive and representative of the diversity of the associated metagenomics sample. We tested this by creating a Kraken (version 2.0.9)^51^ database of sequenced bacterial isolates from a sample and then classifying the reads from the corresponding human fecal metagenome sample. These cultured strains explained most of the metagenomics sequence reads (70%, s.d. = 16%; Extended Data Fig. 1d). Contig building on the remaining bacterial reads did not generate any large contigs; the average length after combining all contigs was only 1.6 million bp, some of which likely resulted from sequencing noise. We also evaluated the comprehensiveness of our cultured bacterial strain library by gavaging several germ-free mice with human stool and performing metagenomics on the mouse fecal samples. The cultured bacterial strains that explained 78% of bacterial reads from the human metagenomics sample explained up to 97% of bacterial reads in the gnotobiotic mice with the same human stool, suggesting that most of the unexplained bacterial metagenomics reads in the human sample were from unculturable sources (for example, dead bacteria from food and environmental sources).

### Reporting Summary

Further information on research design is available in the Nature Research Reporting Summary linked to this article.

## Supplementary information

Reporting Summary

Supplementary Tables Supplementary Tables 1–9 in XLSX workbook format.

## Data Availability

Sequence data files (FASTQ) for all metagenomic sequencing samples are stored in the SRA under project number PRJNA637878. Whole-genome assembled sequences (FASTA) of all the strains have been deposited under project number PRJNA637878. Detailed metadata linking strains and fecal metagenomics to the FMT donor–recipient pair is provided in Supplementary Tables 8 and 9. Source data are provided with this paper.

## Source data

Source Data Fig. 2 Source data for Fig. 2.
Source Data Fig. 3 Source data for Fig. 3.
Source Data Fig. 4 Source data for Fig. 4.
Source Data Extended Data Fig. 1 Source data for Extended Data Fig. 1.
Source Data Extended Data Fig. 2 Source data for Extended Data Fig. 2.
Source Data Extended Data Fig. 3 Source data for Extended Data Fig. 3.

## Code availability

The code for Strainer, a demo application and comparison with other SNP-based strain tracking algorithms is available at https://bitbucket.org/faithj02/strainer-metagenomics/src/master/ and 10.5281/zenodo.5191788.
