# Author Correction: Precise quantification of bacterial strains after fecal microbiota transplantation delineates long-term engraftment and explains outcomes

**DOI:** 10.1038/s41564-022-01118-8

**Published:** 2022-04-06

**Authors:** Varun Aggarwala, Ilaria Mogno, Zhihua Li, Chao Yang, Graham J. Britton, Alice Chen-Liaw, Josephine Mitcham, Gerold Bongers, Dirk Gevers, Jose C. Clemente, Jean-Frederic Colombel, Ari Grinspan, Jeremiah Faith

**Affiliations:** 1 Precision Immunology Institute, Icahn School of Medicine at Mount Sinai, New York, NY USA; 2 Icahn Institute for Data Science and Genomic Technology, Icahn School of Medicine at Mount Sinai, New York, NY USA; 3 Division of Gastroenterology, Icahn School of Medicine at Mount Sinai, New York, NY USA; 4 Janssen Human Microbiome Institute, Janssen Research and Development, LLC, Spring House, PA USA

**Keywords:** Microbiome, Metagenomics

Correction to: *Nature Microbiology* 10.1038/s41564-021-00966-0, published online 27 September 2021.

This paper was originally published under a standard Springer Nature license (© The Author(s), under exclusive licence to Springer Nature Limited). It is now available as an Open Access paper under a Creative Commons Attribution 4.0 International license, © The Author(s). The error has been corrected in the HTML and PDF versions of the article.

