# Supplementary material for: Precise quantification of bacterial strains after fecal microbiota transplantation delineates long-term engraftment and explains outcomes
Source: Nat Microbiol. 2021 Sep 27;6(10):1309–18. doi: 10.1038/s41564-021-00966-0 (PMC8993687; doi:10.1038/s41564-021-00966-0)
Supplement: Supplementary file 1 — Reporting Summary [file 41564_2021_966_MOESM1_ESM.pdf]

nature portfolio

Corresponding author(s): Jeremiah J. Faith

Last updated by author(s): Aug 13, 2021

Reporting Summary

Nature Portfolio wishes to improve the reproducibility of the work that we publish. This form provides structure for consistency and transparency in reporting. For further information on Nature Portfolio policies, see our Editorial Policies and the Editorial Policy Checklist.

Statistics

For all statistical analyses, confirm that the following items are present in the figure legend, table legend, main text, or Methods section.

n/a	Confirmed	
☐	☒	The exact sample size (*n*) for each experimental group/condition, given as a discrete number and unit of measurement
☐	☒	A statement on whether measurements were taken from distinct samples or whether the same sample was measured repeatedly
☐	☒	The statistical test(s) used AND whether they are one- or two-sided<br>*Only common tests should be described solely by name; describe more complex techniques in the Methods section.*
☐	☒	A description of all covariates tested
☐	☒	A description of any assumptions or corrections, such as tests of normality and adjustment for multiple comparisons
☐	☒	A full description of the statistical parameters including central tendency (e.g. means) or other basic estimates (e.g. regression coefficient) AND variation (e.g. standard deviation) or associated estimates of uncertainty (e.g. confidence intervals)
☐	☒	For null hypothesis testing, the test statistic (e.g. *F*, *t*, *r*) with confidence intervals, effect sizes, degrees of freedom and *P* value noted<br>*Give P values as exact values whenever suitable.*
☐	☒	For Bayesian analysis, information on the choice of priors and Markov chain Monte Carlo settings
☐	☒	For hierarchical and complex designs, identification of the appropriate level for tests and full reporting of outcomes
☒	☐	Estimates of effect sizes (e.g. Cohen's *d*, Pearson's *r*), indicating how they were calculated

Our web collection on statistics for biologists contains articles on many of the points above.

Software and code

Policy information about availability of computer code

Data collection	No software was used for data collection.
Data analysis	Code for Strainer, a demo application and comparison with other SNP based strain tracking algorithms is available at https://bitbucket.org/faithj02/strainer-metagenomics/ DOI: 10.5281/zenodo.5191788. We have provided references for all the software used in the manuscript, Trimmomatic version 0.36, KMC version 3.0 and SPADES 3.12. We used Python version 2.7 and R version 3.6.3.

For manuscripts utilizing custom algorithms or software that are central to the research but not yet described in published literature, software must be made available to editors and reviewers. We strongly encourage code deposition in a community repository (e.g. GitHub). See the Nature Portfolio guidelines for submitting code & software for further information.

Data

Policy information about availability of data

All manuscripts must include a data availability statement. This statement should provide the following information, where applicable:

- Accession codes, unique identifiers, or web links for publicly available datasets
- A description of any restrictions on data availability
- For clinical datasets or third party data, please ensure that the statement adheres to our policy

Sequence data files (fastq) for all metagenomic sequencing samples are stored in the public Sequence Read Archive (SRA) under project number PRJNA637878. Whole genome assembled sequences (fasta) of all the strains are also deposited under project number PRJNA637878. Detailed metadata linking strains and faecal metagenomics to the FMT donor-recipient pair is provided in Supplementary Tables 8, 9. All the raw (curated and well annotated data (matched metagenomics samples, gold standard genomic sequence of the cultured bacterial strains, and their unassembled sequenced reads) used for comparison with other strain tracking algorithms have been provided separately at https://bitbucket.org/faithj02/strainer-metagenomics/ DOI: 10.5281/zenodo.5191788.

Field-specific reporting

Please select the one below that is the best fit for your research. If you are not sure, read the appropriate sections before making your selection.

☒ Life sciences ☐ Behavioural & social sciences ☐ Ecological, evolutionary & environmental sciences

For a reference copy of the document with all sections, see nature.com/documents/nr-reporting-summary-flat.pdf

Life sciences study design

All studies must disclose on these points even when the disclosure is negative.

Sample size	This was a longitudinal observational cohort study including all patients 18 years or older with or without IBD who underwent FMT for recurrent or severe CDI between 2013 and 2016 at the Mount Sinai Hospital (New York, USA). We processed all available samples and their future time points. Our algorithm was independently tested on multiple samples, diseases, sequencing read depths, and performs consistently well. The sample sizes were sufficient for our analysis, and all conclusions were independently consistent. We provide confidence intervals on all our estimations of bacterial strain engraftment.
Data exclusions	No data was excluded.
Replication	We analyzed each FMT intervention (n = 13) independently, and our findings replicated uniformly across all independent samples. We tested our strain tracking algorithm on multiple independent disease phenotypes and found consistent performance.
Randomization	All timepoints from a FMT recipient were grouped and analyzed together. Independent FMT interventions were analyzed separately and not grouped together. All subjects received the same therapy. Our results replicated uniformly across all independent samples.
Blinding	This was a longitudinal observational study. Blinding was not relevant as we had access to FMT outcomes and all individuals received the therapy. However our algorithm was developed independently and had consistent performance on all FMT recipient samples.

Reporting for specific materials, systems and methods

We require information from authors about some types of materials, experimental systems and methods used in many studies. Here, indicate whether each material, system or method listed is relevant to your study. If you are not sure if a list item applies to your research, read the appropriate section before selecting a response.

Materials & experimental systems

n/a	Involved in the study	
☒	☐	Antibodies
☒	☐	Eukaryotic cell lines
☒	☐	Palaeontology and archaeology
☐	☒	Animals and other organisms
☐	☒	Human research participants
☒	☐	Clinical data
☒	☐	Dual use research of concern

Methods

n/a	Involved in the study	
☒	☐	ChIP-seq
☒	☐	Flow cytometry
☒	☐	MRI-based neuroimaging

Animals and other organisms

Policy information about studies involving animals; ARRIVE guidelines recommended for reporting animal research

Laboratory animals	Germ free C57BL/6J mice were bred in-house at the Mount Sinai Immunology Institute Gnotobiotic facility in flexible vinyl isolators. Shortly after weaning (28-42 days old) and under strict aseptic conditions, germ-free mice were transferred to autoclaved filter-top cages outside the breeding isolator and colonized with human microbiotas. Mice were colonized with 200-300 ul of a fecal slurry or pooled cocktail of cultured strains by oral gavage, given only once.
Wild animals	Study did not involve wild animals.
Field-collected samples	Study did not involve samples collected from the field.
Ethics oversight	All animal studies were carried out in accordance with protocols approved by the Institutional Animal Care and Use Committee (IACUC) in Icahn School of Medicine at Mount Sinai.

Note that full information on the approval of the study protocol must also be provided in the manuscript.

Human research participants

Policy information about studies involving human research participants

Population characteristics	The original longitudinal cohort study had 134 patients with CDI, of which 64% were women and average age was 53 years. 46 patients also had IBD, and among these patients 27 had ulcerative colitis (UC), 18 had Crohn's disease (CD) and 1 had indeterminate colitis. Average age of patients with IBD was 38.8 ± 20.5 years and those with non-IBD was 60.3 ± 18.9 years. 54.3% of patients with IBD, and 69.3% of non-IBD patients were women. All human research participants have been described in greater detail in a previously published study (and cited in the manuscript).
Recruitment	This was a longitudinal cohort study including all patients 18 years or older with or without IBD who underwent FMT for recurrent or severe CDI between 2013 and 2016 at the Mount Sinai Hospital (New York, USA). Eligibility criteria for FMT at our institution included recurrent CDIs characterized as (1) at least 3 episodes of mild to moderate CDI and failure of a 6- to 8-week taper with vancomycin and (2) at least 2 episodes of severe CDI resulting in hospitalizations and associated with significant morbidity. Eligibility also included severe CDIs characterized as (1) persistent moderate to severe CDI not responding to standard therapy (vancomycin) for at least 1 week and (2) severe (including fulminant) CDI with no response to standard therapy after 48 hours. The recruitment criteria has been described in greater detail in a previously published study (and cited in the manuscript) on this observational cohort. Potential self-selection bias is a possibility as only a subset of patients consistently provided their stool samples at multiple future time-points post FMT. This is not likely to impact our results as we have both responders and non-responders in our cohort.
Ethics oversight	Mount Sinai Institutional Review Board (HS# 11-01669) approved the study protocol. All the participants provided their written consent.

Note that full information on the approval of the study protocol must also be provided in the manuscript.
